# Gender and pandemic perception: analyzing perceived risk and fear among Brazilian women

**DOI:** 10.1080/21642850.2021.1968301

**Published:** 2021-09-06

**Authors:** Rubia Carla Formighieri Giordani, Suely Ruiz Giolo, Milene Zanoni da Silva, Camila Muhl

**Affiliations:** a Department of Nutrition, Federal University of Parana, Curitiba, Brazil; b Department of Statistics, Federal University of Parana, Curitiba, Brazil; c Department of Collective Health, Federal University of Parana, Curitiba, Brazil; d Department of Psychology, FAE University, Curitiba, Brazil

**Keywords:** Gender, pandemics, risk perception, severity, susceptibility

## Abstract

**Objective:**

The World Health Organization has warned of the importance of looking at the pandemic from a gender perspective since biological, social, and cultural aspects can produce differences in the way women and men experience the ongoing pandemic situation. This study aimed to investigate Brazilian women’s perception of COVID-19 concerning the susceptibility to infection, the severity of COVID-19, and the collective behavior in response to pandemic risks. It also sought to examine the COVID-19 level of perceived fears by the level of fear across specific COVID-19 fears, such as the risk of infection and the threat posed to life.

**Methods:**

The sample consisted of 5527 Brazilian women aged between 18 and 74 years (mean age = 41, SD = 13.5) recruited from an online cross-sectional survey conducted in Brazil. The analysis addressed questions about the chance of COVID-19 infection, the impact on daily activities in case of infection, the threat to life posed by COVID-19, and the collective behavior in the pandemic context. In addition, the fear of COVID-19 was assessed using the Fear of COVID-19 Scale (FCV-19S).

**Results:**

Women’s age and personal conditions, particularly those related to family and domestic life, showed association with the perceived susceptibility to infection and the severity of COVID-19. The higher the perceived susceptibility and severity, the greater the fear level. The fear level was also higher among women whose perception was that people were ignoring the pandemic risks.

**Conclusion:**

This study provides insight into Brazilian women’s perception of COVID-19 and their fear level during the pandemic’s early stages. Our findings can increase the understanding of the adverse consequences caused by the COVID-19 pandemic on women, assisting in the strategic planning of public policies.

## 1. Introduction

The Sociology of risk, developed by authors such as Anthony Giddens and Ulrich Beck, understands that risk perception influences people’s actions in the world. Risk is the assessment of negative results that may arise due to human actions or activities and is related to the feeling of security and trust when it is minimized from the belief in a person’s credibility or a system. On the other hand, when the risk is experienced, it brings the perception of danger and fear (Giddens, 1991).

In addition, emotions play a critical role in the perception of risk itself (Bavel et al., 2020), also influencing the cognitive evaluations of risk and triggering responses to risk situations (Loewenstein, Hsee, Weber, & Welch, 2001). When carrying out the risk assessment of a situation, fear arises in the face of the anticipation of unfavorable results and the threat to ontological security. This feeling of threat to ontological security hurts the tacit belief that every human being has in his identity, as well as in the assured continuity of the social spaces that constitute his world (Giddens, 1991).

Considering that, for the Sociology of risk, the perception of risk goes through social processes of definition and that there are social determinants of health and disease processes (which includes perceptions and assessments produced by individuals’ specific social conditions), it is assumed that life situations generate particular experiences of the pandemic.

It is also worth noting that, although the pandemic has affected populations worldwide, it is evident that social inequalities in health are unevenly impacting COVID-19 morbidity and mortality (Abrams & Szefler, 2020). Concerning exposure to the virus, vulnerability to infection and consequences of the disease (including psychological, social, and economic effects), social markers (income and educational level) are determinants for worse outcomes (Burstrom & Tao, 2020). In countries without public health systems, income can be an indicator of accessibility to health care. It is also noteworthy that in low- and middle-income countries where many people work informally and with job insecurity, a large part of the population cannot ‘stay at home.’ Due to the low capacity of local governments to implement minimum income relief measures and appropriate public policies for the most socially vulnerable populations, poverty has become an important barrier in the adherence to non-pharmacological measures.

Since the beginning of the global health crisis caused by the COVID-19 pandemic, the World Health Organization has warned of the importance of considering that men and women are affected differently by the pandemic emergency in its multiple aspects (WHO, n.d.).

Specifically concerning mental health, the results of a study carried out in Italy indicated greater psychological vulnerability to the pandemic among women (Rossi et al., 2020). However, even if it is considered that women are usually more likely to develop anxiety disorders and specific phobias (Kuehner, 2017), the COVID-19 pandemic evidenced how a biological variable becomes a powerful social marker so that biological differences can become health inequities. In the current pandemic, differences in life trajectories are being produced by social, cultural, and economic factors, and they are acting as conditioning factors for gender differences (Gausman & Langer, 2020).

The social and economic consequences of the pandemic have disproportionately affected women (WHO, n.d.). In low- and middle-income countries, women were most affected by measures to control the COVID-19 pandemic in several aspects (Alon, Doepke, Olmstead-Rumsey, & Tertilt, 2020; Parry & Gordon, 2021). For example, among the most economically vulnerable female segments, there was a decrease or loss of income due to the interruption of paid activities related to domestic work (such as cleaning and caring for children and the elderly) and restriction of access and circulation in public spaces, where they exercised informal activities (Wenham, Smith, & Morgan, 2020).

In addition to the quarantine’s adverse effects, which for the population as a whole are characterized by anxiety, depression, and panic disorders (Brooks et al., 2020), there was an expressive increase in domestic violence cases against women. (Hamadani et al., 2020; WHO, n.d.). Thus, the adverse emotional effects of confinement, exposure and prolonged coexistence with an abusive and aggressive partner, enhanced by the difficulty of accessing shelters and reporting channels and the financial instability and dependence, have considerably worsened the female condition during the pandemic.

Also, access to sexual and reproductive health rights may have been reduced during the pandemic for women and girls. For example, pregnant and parturient women may not be receiving adequate medical care or may be deprived of partners or doulas, mainly due to the new health protocols and the overload of medical services (Gausman & Langer, 2020).

In other words, social determinants (such as gender, age, education level, and income) condition exposure to the virus, but they also generate specific situations that modulate emotions and produce feelings of greater or lesser danger that imply perception and assessment of risk.

Furthermore, the World Health Organization has been advised countries to use a gender-based approach in their responses to the COVID-19 pandemic. The WHO has recommended that public health policies and measures to mitigate the pandemic consider gender from an intersectional perspective to address health inequalities (any observable differences between subgroups) and health inequities (differences considered unfair based on a value judgment) (Arcaya, Arcaya, & Subramanian, 2015; Ryan & El Ayadi, 2020; WHO, 2013, 2015, n.d.). It should also be noted that although studies on COVID-19 and gender have addressed the unequal impact of the pandemic on the lives of women and men, studies aimed at identifying the most vulnerable groups among women are still a knowledge gap. In this sense, intersectionality as a concept that highlights that different statuses are attributed to people as a function of race, gender, social class, age, among other social markers, favors the understanding of differences between women (Hooks, 2015).

Based on these assumptions, this study used fear, perceived susceptibility to infection, and perceived severity of COVID-19 as indicators of risk perception, as initially formulated. Thus, this study aimed to (1) describe women’s perceptions of COVID-19 fears (as measured by FCV-19S items); (2) examine COVID-19 level of perceived fears (as measured by the FCV19S) by level of fear across specific COVID-19 fears (i.e. the chance of infection, the threat posed to life, impact on daily activities, and other people’s behavior); and (3) examine sociodemographic and family/domestic life correlates of women’s perceived susceptibility to COVID-19 infection, perceived impact of COVID-19 infection on daily activities; perceived threat to life posed by COVID-19, and perceived collective behavior in response to pandemic risks.

## 2. Material and methods

### 2.1. Participants and procedures

A cross-sectional online study based on a convenience sample was conducted with Brazilian women aged 18–74 years (mean age = 41; SD = 13.5) residing in Brazil. The majority were in the age group of 30–59 years (66.6%), had higher education (90.8%), and were from the Brazilian capital cities (59.2%). Also, 32.1% had an average monthly family income between 5 and 10 national minimum wages and 23% above 10. Brazil’s minimum monthly wage is currently around 204 USD. In addition, a significant part (73.6%) had private health insurance, and 58.3% had people at home with medical conditions at increased risk for severe illness from COVID-19 (such as cardiovascular disease, diabetes, chronic respiratory diseases, and cancer). The data were collected from July 1–20, 2020, using an electronic survey disseminated through social media, WhatsApp, Facebook, and institutional public services email (universities and the judiciary). Participation was voluntary. All participants provided electronic informed consent. The Research Ethics Committee of the Federal University of Parana approved the study (CAAE 32823220.7.0000.0102).

### 2.2. General data

The questionnaire developed on the JotForm® platform included sociodemographic questions (age, family income, educational level, and place of residence) and multiple-choice questions related to family and domestic life (e.g. children at home under ten years of age, people at home with medical conditions at increased risk for severe illness from COVID-19, and people at home who are health professionals). It also included the seven items of the Fear of COVID-19 Scale (FCV-19S) and multiple-choice questions related to women’s perception of COVID-19.

### 2.3. Measures

Following the sociological notion of risk as a basis for investigating women’s perception of COVID-19, the following outcomes were used as indicators of risk perception: fear of COVID-19 (assessed using the FCV-19 Scale), perceived susceptibility to infection (assessed through a question about the chance of infection), perceived severity of COVID-19 (assessed through questions about the threat to life posed by COVID-19 and the impact on daily activities in case of infection), and perceived collective behavior in response to pandemic risks (assessed through a question about whether or not people are ignoring the pandemic risks).

#### 2.3.1. The fear of COVID-19 Scale (FCV-19S)

The FCV-19S is a seven-item scale developed by Ahorsu et al. (2020) to assess the fear of COVID-19. The items of the FCV-19S were constructed based on an extensive review of existing scales on fears, expert evaluations, and participant interviews. An example item of the FCV-19S is ‘ I am afraid of losing my life because of coronavirus.’ Table 1 shows the seven items included in the online survey. All items of the FCV-19S were answered on a five-point Likert scale ranging from 1 to 5 (strongly disagree, disagree, neither agree nor disagree, agree and strongly agree). The overall score of fear corresponds to the seven items’ sum and varies from 7 to 35. The higher the score, the greater the fear of COVID-19. Cavalheiro and Sticca (2020) and Giordani, Giolo, Muhl, and Zanoni da Silva (2021) validated the Portuguese version of the FCV-19S in Brazil. Its internal consistency reliability (Cronbach’s alpha of 0.88 and 0.86 reported by the mentioned authors) was consistent with the reliability of 0.82 reported by Ahorsu et al. (2020). It also showed a unidimensional factor structure and robust psychometric properties to measure fear of COVID-19 in Brazil’s general population.

**Table 1.** Item distribution of the FCV-19S responses, item mean scores, and standard deviation (SD).

Items	Description of the FCV-19S items	Number (%)					Mean (SD)
		1	2	3	4	5	
Item 1	I am most afraid of the coronavirus	120 (2.2)	582 (10.5)	1321 (23.9)	2434 (44.0)	1070 (19.4)	3.68 (0.97)
Item 2	It makes me uncomfortable to think about coronavirus	148 (2.7)	769 (13.9)	1163 (21.1)	2588 (46.8)	859 (15.5)	3.58 (0.99)
Item 3	My hands become clammy when I think about coronavirus	1617 (29.3)	2708 (49.0)	737 (13.3)	372 (6.7)	93 (1.7)	2.02 (0.92)
Item 4	I am afraid of losing my life because of coronavirus	353 (6.4)	1047 (18.9)	1171 (21.2)	2282 (41.3)	674 (12.2)	3.34 (1.11)
Item 5	When watching news and stories about coronavirus on social media, I become nervous or anxious	311 (5.6)	1049 (19.0)	1121 (20.3)	2306 (41.7)	740 (13.4)	3.38 (1.10)
Item 6	I cannot sleep because I’m worried about getting coronavirus	1554 (28.1)	2659 (48.1)	852 (15.4)	398 (7.2)	64 (1.2)	2.05 (0.91)
Item 7	My heart races or palpitates when I think about coronavirus	1239 (22.4)	2380 (43.1)	898 (16.2)	841 (15.2)	169 (3.1)	2.33 (1.07)

Note: Each item was scored on a five-point Likert scale where score 1 = strongly disagree to score 5 = strongly agree. The seven items described in the table correspond to the items of the FCV-19 Scale developed by Ahorsu et al. (2020).

#### 2.3.2. Perceived susceptibility to COVID-19 infection

The perceived susceptibility to COVID-19 infection was assessed using the following question: ‘My chance of being infected with COVID-19 is … ’ The question was answered on a five-point Likert scale ranging from very unlikely to very likely. The two levels considered for the association analysis were: high (very likely or likely) and low (neither likely nor unlikely, unlikely or very unlikely). The grouping on two levels was due to the low frequencies for some categories resulting from crossing this variable with others in the association analysis.

#### 2.3.3. Perceived severity of COVID-19: the threat to life posed by COVID-19

The first question asked to assess the perceived severity of COVID-19 was: ‘The disease caused by COVID-19 is … ’ The response options were: it is a threat to life because it is contagious and has no treatment; it is not dangerous because I am not part of the high-risk groups, and I do not know. The severity levels considered for the association analysis were: high (it is a threat to life) and low (it is not dangerous, or I do not know).

#### 2.3.4. Perceived severity of COVID-19: impact on daily activities in case of infection

Most people infected with COVID-19 will experience mild to moderate respiratory illness and recover without hospitalization, while others will develop severe illness, requiring hospitalization. Thus, in addition to the question about the threat to life posed by COVID-19, another question asked to assess the perceived severity of COVID-19 was about the impact on daily activities in case of COVID-19 infection. It was: ‘If I become infected, the chance of not maintaining my daily activities is … ’ The responses were on a five-point Likert scale ranging from very unlikely to very likely. The levels considered for the association analysis were: high (very likely or likely) and low (neither likely nor unlikely, unlikely, or very unlikely). The grouping on two levels was due to the low frequencies for some categories resulting from crossing this variable with others in the association analysis.

#### 2.3.5. Perceived collective behavior in response to pandemic risks

The question asked to assess the women’s perception of other people’s behavior in response to pandemic risks was: ‘In my perception, the people around me are … ’ The response options were: insensitive to the pandemic and ignoring its risks, overreacting to pandemic risks, and neither overly frightened nor ignoring the pandemic risks. For this question, the levels considered for the association analysis were: ignoring the risks (insensitive to the pandemic and ignoring its risks) and not ignoring the risks (neither overly frightened nor ignoring the pandemic risks, and overreacting to pandemic risks).

According to the theoretical assumptions explained in the introduction, the risk is the perception or evaluation of negative results, and fear is a feeling linked to the experience of risk. Consequently, fear generates a response in the perception and assessment of risk (represented in our study by the perceived risk of COVID-19 infection, perceived threat to life, and perceived impact on daily activities in case of infection). In addition, the perception of other people’s behavior in response to pandemic risks generates an emotional response of fear.

### 2.4. Statistical analysis

Analyses included conducting descriptive statistics of FCV-19S items to describe women’s perceptions of COVID-19 fears (Table 1). In addition, to examine COVID-19 level of perceived fears (measured by FCV-19S) by level of fear across specific COVID-19 fears (i.e. the chance of infection, the threat posed to life, impact on daily activities, and other people’s behavior), analyzes included conducting descriptive statistics, one-way analysis of variance, and Tukey's HSD post hoc test (Table 2). Finally, to examine sociodemographic and family/domestic life correlates of women’s perceived susceptibility to COVID-19 infection (i.e. risk of high infection: Table 3), perceived impact of COVID-19 infection on daily activities (i.e. risk of high impact: Table 4), perceived threat to life posed by COVID-19 (i.e. risk of a high level of threat: Table 5), and perceived collective behavior of ignoring pandemic risks (Table 6), analyses included conducting multiple logistic regressions separately for each outcome. The adjusted odds ratio (OR) and its respective 95% confidence intervals were used to discuss the variables’ association. Binary logistic regression was used instead of ordinal logistic regression due to the low frequencies observed for some response categories. All analyzes were performed using the R software (R Core Team, 2020).

**Table 2.** Comparison of the overall mean fear scores of COVID-19 (measured from the FCV-19S scores).

Variable	*N* (%)	Mean score of fear	
		Mean ± SD	*P*-value
Chance of COVID-19 infection			
Very unlikely	66 (1.2)	18.4 ± 6.2 (a)	0.92
Unlikely	344 (6.2)	19.0 ± 5.7 (a)	
Neither likely nor unlikely	2159 (39.1)	20.1 ± 5.0 (b)	< 0.01
Likely	2290 (41.4)	20.6 ± 5.1 (c)	
Very likely	668 (12.1)	21.4 ± 5.6 (d)	
The threat posed by COVID-19			
It is a threat to life	4270 (77.3)	21.3 ± 4.9 (a)	< 0.001
It is not dangerous	578 (10.4)	16.0 ± 5.0 (b)	
I do not know	679 (12.3)	18.3 ± 4.6 (c)	
Impact on daily activities in case of infection			
Very unlikely	168 (3.0)	19.4 ± 6.4 (a)	0.97
Unlikely	599 (10.8)	19.7 ± 5.4 (a)	
Neither likely nor unlikely	938 (17.0)	19.7 ± 5.1 (a)	
Likely	1816 (32.9)	19.9 ± 5.0 (a)	
Very likely	2006 (36.3)	21.3 ± 5.2 (b)	<0.01
Perception of people’s behavior			
Insensitive to the pandemic and ignoring its risks	2740 (49.6)	21.6 ± 5.1 (a)	< 0.001
Overreacting to pandemic risks	194 (3.5)	15.9 ± 6.2 (b)	
Neither overly frightened nor ignoring	2593 (46.9)	19.5 ± 4.9 (c)	
Overall	5527 (100)	20.4 ± 5.2	–

Note: According to the Tukey HSD test, different letters between two categories (levels) of a variable indicate a significant difference in their respective mean scores at *p* < .01 or *p* < .001. The opposite indicates no significant difference.

**Table 3.** Multiple logistic regression of the perceived susceptibility to COVID-19 infection.

		Chance of COVID-19 infection		
Variable	*N* (%)	High *N* (%)	Low *N* (%)	Odds ratio (95%CI)
Age group				
18–29 years	1264 (22.9)	683 (54.0)	581 (46.0)	Reference
30–59 years	3681 (66.6)	2077 (56.4)	1604 (43.7)	1.06 (0.93–1.21)^NS^
60 years and above	582 (10.5)	198 (34.0)	384 (66.0)	0.45 (0.36–0.57)**
Average monthly family income				
Up to 1 national minimum wage	208 (3.8)	96 (46.2)	112 (53.8)	0.77 (0.56–1.07)^NS^
From 1 to 2	613 (11.1)	327(53.3)	286 (46.7)	Reference
From 2 to 5	1775 (32.1)	972 (54.7)	803 (45.3)	1.03 (0.84–1.24)^NS^
From 5 to 10	1657 (30.0)	881 (53.2)	776 (46.8)	0.98 (0.80–1.20)^NS^
Above 10	1274 (23.0)	682 (53.5)	592 (46.5)	1.05 (0.84–1.31)^NS^
Education level				
Basic education	40 (0.7)	20 (50.0)	20 (50.0)	Reference
High school	471 (8.5)	217 (46.1)	254 (53.9)	0.75 (0.38–1.46)^NS^
Higher education	5016 (90.8)	2721 (54.3)	2295 (45.7)	0.98 (0.51–1.87)^NS^
The city of residence				
State capital city	3273 (59.2)	1715 (52.4)	1558 (47.6)	Reference
Up to 50,000 inhabitants	597 (10.8)	312 (52.3)	285 (47.7)	0.95 (0.80–1.14)^NS^
50,000–100,000	379 (6.9)	211 (55.7)	168 (44.3)	1.11 (0.89–1.40)^NS^
Over 100,000 inhabitants	1278 (23.1)	720 (56.3)	558 (43.7)	1.12 (0.98–1.28)^NS^
Children aged under 10				
No	4187 (75.7)	2176 (52.0)	2011 (48.0)	Reference
Yes	1340 (24.3)	782 (58.3)	558 (41.6)	1.16 (1.02–1.32)**
Resident aged 65 years or older				
No	4435 (80.3)	2426 (54.7)	2009 (45.3)	Reference
Yes	1092 (19.8)	532 (48.7)	560 (51.3)	0.89 (0.77–1.04)^NS^
Residents with high-risk medical conditions for COVID-19				
No	2308 (41.7)	1186 (51.4)	1122 (48.6)	Reference
Yes	3219 (58.3)	1772 (55.1)	1447 (44.9)	1.21 (1.08–1.36)**
Has a private health insurance				
No	1461 (26.4)	793 (54.3)	668 (45.7)	Reference
Yes	4066 (73.6)	2165 (53.3)	1901 (46.7)	0.93 (0.81–1.07)^NS^
Living with a health professional				
No	3972 (71.9)	1994 (50.2)	1978 (49.8)	Reference
Yes	1555 (28.1)	964 (62.0)	591 (38.0)	1.57 (1.39–1.77)**
Overall	5527 (100)	2958 (53.5)	2569 (46.5)	–

Note: Brazil’s minimum monthly wage is currently 204 USD; CI: confidence interval; NS: non-significant; *p < .05 and **p < .001.

**Table 4.** Multiple logistic regression of the perceived severity of COVID-19 (impact on daily activities).

		Impact on daily activities in case of COVID-19 infection		
Variable	*N* (%)	High	Low	Odds ratio
		*N* (%)	*N* (%)	(95%CI)
Age group				
18–29 years	1264 (22.9)	812 (64.3)	452 (35.7)	Reference
30–59 years	3681 (66.6)	2599 (70.6)	1082 (29.4)	1.34 (1.16–1.55)**
60 years and above	582 (10.5)	411 (70.6)	171 (29.4)	1.32 (1.05–1.66)**
Average family income				
Up to 1 minimum wage	208 (3.8)	138 (66.3)	70 (33.7)	1.06 (0.76–1.49)^NS^
From 1 to 2	613 (11.1)	404 (65.9)	209 (34.1)	Reference
From 2 to 5	1775 (32.1)	1231 (69.3)	544 (30.6)	1.09 (0.89–1.34)^NS^
From 5 to 10	1657 (30.0)	1138 (68.7)	519 (31.3)	1.02 (0.82–1.27)^NS^
Over 10	1274 (23.0)	911 (71.5)	363 (28.5)	1.15 (0.91–1.45)^NS^
Education level				
Basic education	40 (0.7)	27 (67.5)	13 (32.5)	Reference
High school	471 (8.5)	304 (64.5)	167 (35.5) 1525 (30.4)	0.89 (0.43–1.75)^NS^
Higher education	5016 (90.8)	3491 (69.6)		1.06 (0.52–2.04)^NS^
The city of residence				
State capital city	3273 (59.2)	2280 (69.7)	993 (30.3)	Reference
Up to 50,000 inhabitants	597 (10.8)	394 (66.0)	203 (34.0)	0.87 (0.72–1.06)^NS^
50,000–100,000	379 (6.9)	271 (71.5)	108 (28.5)	1.11 (0.88–1.41)^NS^
Over 100,000 inhabitants	1278 (23.1)	877 (68.6)	401 (31.4)	0.95 (0.82–1.09)^NS^
Children aged under 10				
No	4187 (75.7)	2893 (69.1)	1294 (30.9)	Reference
Yes	1340 (24.3)	929 (69.3)	411 (30.7)	0.98 (0.85–1.13)^NS^
Residents aged 65 years or older				
No	4435 (80.3)	3058 (68.9)	1377 (31.1)	Reference
Yes	1092 (19.8)	764 (70.0)	328 (30.0)	0.94 (0.80–1.10)^NS^
Residents with high-risk medical conditions for COVID-19				
No	2308 (41.7)	1530 (66.3)	778 (33.7)	Reference
Yes	3219 (58.3)	2292 (71.2)	927 (28.8)	1.27 (1.13–1.44)**
Has a private health insurance				
No	1461 (26.4)	994 (68.0)	467 (32.0)	Reference
Yes	4066 (73.6)	2828 (69.6)	1238 (30.4)	0.97 (0.83–1.12)^NS^
Living with a health professional				
No	3972 (71.9)	2712 (68.3)	1260 (31.7)	Reference
Yes	1555 (28.1)	1110 (71.4)	445 (28.6)	1.13 (0.99–1.29)^NS^
Overall	5527 (100)	3822 (69.2)	1705 (30.8)	–

Note: Brazil’s minimum monthly wage is currently 204 USD; CI: confidence interval; NS: non-significant; and **p < .001.

**Table 5.** Multiple logistic regression of the perceived severity of COVID-19 (threat to life posed by COVID-19).

		The threat to life posed by COVID-19		
Variable	*N* (%)	High	Low	Odds ratio
		*N* (%)	*N* (%)	(95%CI)
Age group				
18–29 years	1264 (22.9)	905 (71.6)	359 (28.4)	Reference
30–59 years	3681 (66.6)	2867 (77.9)	814 (22.1)	1.58 (1.35–1.84)**
60 years and above				
	582 (10.5)	498 (85.6)	84 (14.4)	2.45 (1.86–3.27)**
Average monthly family income				
Up to 1 national minimum wage	208 (3.8)	148 (71.2)	60 (28.8)	0.84 (0.57–1.21)^NS^
From 1 to 2	613 (11.1)	463 (75.5)	150 (24.5)	Reference
From 2 to 5	1775 (32.1)	1389 (78.2)	386 (21.7)	1.06 (0.85–1.34)^NS^
From 5 to 10	1657 (30.0)	1280 (77.3)	377 (22.7)	0.97 (0.76–1.23)^NS^
Over 10	1274 (23.0)	990 (77.7)	284 (22.3)	0.97 (0.75–1.26)^NS^
Education level				
Basic education	40 (0.7)	32 (80.0)	8 (20.0)	Reference
High school	471 (8.5)	345 (73.3)	126 (26.7)	0.71 (0.29–1.54)^NS^
Higher education	5016 (90.8)	3893 (77.6)	1123 (22.4)	0.85 (0.35–1.81)^NS^
The city of residence				
State capital city	3273 (59.2)	2560 (78.2)	713 (21.8)	Reference
Up to 50,000 inhabitants	597 (10.8)	435 (72.9)	162 (27.1)	0.87 (0.72–1.05)^NS^
50,000–100,000	379 (6.9)	297(78.4)	82 (21.6)	0.99 (0.76–1.30)^NS^
Over 100,000 inhabitants	1278 (23.1)	978 (76.5)	300 (23.5)	0.91 (0.78–1.07)^NS^
Children aged under 10				
No	4187 (75.7)	3283 (78.4)	904 (21.6)	Reference
Yes	1340 (24.3)	987 (73.7)	353 (26.3)	0.78 (0.67–0.90)**
Residents aged 65 years or older				
No	4435 (80.3)	3390 (76.4)	1045 (23.6)	Reference
Yes	1092 (19.8)	880 (80.6)	212 (19.4)	1.26 (1.05–1.51)**
Residents with high-risk medical conditions for COVID-19				
No	2308 (41.7)	1589 (68.8)	719 (31.2)	Reference
Yes	3219 (58.3)	2681 (83.3)	538 (16.7)	2.39 (2.09–2.74)**
Has a private health insurance				
No	1461 (26.4)	1119 (76.6)	342 (23.4)	Reference
Yes	4066 (73.6)	3151 (77.5)	915 (22.5)	0.92 (0.78–1.09)^NS^
Living with a health professional				
No	3972 (71.9)	3089 (77.8)	883 (22.2)	Reference
Yes	1555 (28.1)	1181 (75.9)	374 (24.1)	0.86 (0.74–1.01)^NS^
Overall	5527 (100)	4270 (77.3)	1257 (22.7)	–

Note: Brazil’s minimum monthly wage is currently 204 USD; CI: confidence interval; NS: non-significant; and **p < .001.

**Table 6.** Multiple logistic regression of the perceived collective behavior in response to pandemic risks

Variable		Other people’s behavior in response to pandemic risks		
	*N* (%)	Ignoring	Not ignoring	Odds ratio
		*N* (%)	*N* (%)	(95%CI)
Age group				
18–29 years	1264 (22.9)	807 (63.8)	457 (36.2)	Reference
30–59 years	3681 (66.6)	1752 (47.6)	1929 (52.4)	0.56 (0.48–0.64)**
60 years and above	582 (10.5)	181 (31.1)	401 (68.9)	0.29 (0.23–0.36)**
Average monthly family income				
Up to 1 national minimum wage	208 (3.8)	124 (59.6)	84 (40.4)	1.06 (0.76–1.47)^NS^
From 1 to 2	613 (11.1)	358 (58.4)	255 (41.6)	Reference
From 2 to 5	1775 (32.1)	946 (53.3)	829 (46.7)	0.86 (0.71–1.05)^NS^
From 5 to 10	1657 (30.0)	778 (47.0)	879 (53.0)	0.73 (0.59–0.90)**
Over 10	1274 (23.0)	534 (41.9)	740 (58.1)	0.65 (0.52–0.81)**
Education level				
Basic education	40 (0.7)	20 (50.0)	20 (50.0)	Reference
High school	471 (8.5)	220 (46.7)	251 (53.3)	0.75 (0.38–1.45)^NS^
Higher education	5016 (90.8)	2500 (49.8)	2516 (50.2)	1.03 (0.54–1.98)^NS^
The city of residence				
State capital city	3273 (59.2)	1576 (48.2)	1697 (51.8)	Reference
Up to 50,000 inhabitants	597 (10.8)	298 (49.9)	299 (50.1)	0.92 (0.77–1.11)^NS^
50,000–100,000	379 (6.9)	209 (55.2)	170 (44.8)	1.16 (0.93–1.44)^NS^
Over 100,000 inhabitants	1278 (23.1)	657 (51.4)	621 (48.6)	1.09 (0.95–1.24)^NS^
Children aged under 10				
No	4187 (75.7)	2058 (49.2)	2129 (50.8)	Reference
Yes	1340 (24.3)	682 (50.9)	658 (49.1)	1.11 (0.97–1.26)^NS^
Residents aged 65 years or older				
No	4435 (80.3)	2221 (50.1)	2214 (49.9)	Reference
Yes	1092 (19.8)	519 (47.5)	573 (52.5)	0.99 (0.86–1.15)^NS^
Residents with high-risk medical conditions for COVID-19				
No	2308 (41.7)	1041 (45.1)	1267 (54.9)	Reference
Yes	3219 (58.3)	1699 (52.8)	1520 (47.2)	1.37 (1.22–1.54)**
Has a private health insurance				
No	1461 (26.4)	810 (55.4)	651 (44.6)	Reference
Yes	4066 (73.6)	1930 (47.5)	2136 (52.5)	0.92 (0.80–1.06)^NS^
Living with a health professional				
No	3972 (71.9)	1977 (49.8)	1995 (50.2)	Reference
Yes	1555 (28.1)	763 (49.1)	792 (50.9)	0.96 (0.85–1.08)^NS^
Overall	5527 (100)	2740 (49.6)	2787 (50.4)	–

Note: Brazil’s minimum monthly wage is currently 204 USD; CI: confidence interval; NS: non-significant; and **p < .001.

## 3. Results

### 3.1. The fear of COVID-19

Based on the responses to the FCV-19S items, Table 1 shows that 63.4% of women reported a high level of fear of the coronavirus. Also, 53.5% were afraid of losing their lives because of the coronavirus. Regarding the item’s mean scores, the two highest were related to item 1 [‘I am most afraid of the coronavirus’] (3.68, SD = 0.97) and item 2 [‘It makes me uncomfortable to think about coronavirus’] (3.58, SD = 0.99).

Results in Table 2 show that 53.5% stated that the chance of COVID-19 infection is likely or very likely, and 77.3% considered COVID-19 a real threat to life. Moreover, 69.2% reported that the impact on daily activities if infected is likely or very likely, and 49.6% reported that people were insensitive to the pandemic and ignoring its risks. Higher mean fear scores were associated with women who reported that people were ignoring the pandemic risks (21.6 ± 5.1), that COVID-19 infection is very likely (21.4 ± 5.6), that COVID-19 represents a high threat to life (21.3 ± 4.9), and that the impact on daily life in case of COVID-19 infection is very likely (21.3 ± 5.2).

### 3.2. Perceived susceptibility to infection

The multiple logistic regression results displayed in Table 3 reveal that family income, education level, city of residence, elderly family members, and private health insurance had no significant association with the women’s perceived susceptibility to COVID-19 infection. Women who mainly perceived high susceptibility to infection had children under ten years of age, had people at home with high-risk medical conditions for severe illness from COVID-19, and lived with health professionals. Compared to their respective reference groups, these women showed an increase of 16%, 21%, and 57%, respectively, in the odds of perceiving a high susceptibility to COVID-19 infection. Compared to women aged 60 years and above, those aged 18–29 and 30–59 years also showed a 122% increase in the odds of perceiving themselves as more susceptible to COVID-19 infection (OR = 1/0.45 = 2.22).

### 3.3. Perceived severity of COVID-19: impact on daily activities in case of infection

Compared to women aged 18–29, Table 4 shows that women aged 30–59 and ≥ 60 years presented an increase of 34% and 32%, respectively, in the odds of perceiving a high impact on daily activities in case of infection. Also, women who had people at home with high-risk medical conditions for severe illness from COVID-19 showed a 27% increase in the mentioned odds than those who did not. Variables such as family income, education level, city of residence, and private health insurance showed no significant association with the perceived impact on daily activities in case of infection (Table 4).

### 3.4. Perceived severity of COVID-19: the threat to life posed by COVID-19

Table 5 shows that the perceived severity level of COVID-19 is strongly associated with women’s age. Compared to women aged 18–29 years, those aged 30–59 and ≥ 60 years showed an increase of 58% and 145%, respectively, in the odds of perceiving COVID-19 as a high threat to life. In addition, women who had people at home with high-risk medical conditions or elderly in the family (aged ≥ 65) showed an increase of 139% and 26%, respectively, in the mentioned odds compared to those who did not. Variables such as family income, education level, city of residence, and private health insurance showed no significant association with the perceived threat to life posed by COVID-19.

### 3.5. Perceived collective behavior in response to pandemic risks

According to Table 6, the women who most perceived other people’s behavior as ignoring the pandemic risks were those aged 18–29 years, who had an average monthly family income of less than five national minimum wages, and who had residents with high-risk medical conditions for COVID-19. For example, compared to women aged 60 and over, those aged 18–29 showed an increase of 245% in the odds of perceiving other people’s behavior as ignoring the pandemic risks. On the other hand, education and city of residence, amongst others, showed a non-significant association with perceived collective behavior in response to pandemic risks (Table 6).

## 4. Discussion

The primary purpose of this study was to understand some aspects of Brazilian women’s perception about the COVID-19 pandemic, orienting the analysis of the results from a perspective that considers the particularities that constitute being a woman. These particularities produced by different living conditions define the pandemic’s emotional experience and the assessment and perception of risk.

In our study, women showed a high-risk perception of the COVID-19 pandemic, in which 53.5% perceived high susceptibility to infection, 77.3% considered that COVID-19 represents a high threat to life, 69.2% perceived a high impact on daily activities in case of infection, and 63.4% had higher levels of fear. Among the most vulnerable subgroups of Brazilian women, concerning the perceived susceptibility to COVID-19 infection, are those having children under the age of ten, having people at home with high-risk medical conditions for severe illness from COVID-19, living with health professionals, and under the age of 60 years. Concerning the severity of COVID-19, women aged 30–59 and ≥ 60 years perceived a higher impact on daily activities in case of COVID-19 infection than younger women did. Also, the high threat posed by COVID-19 to life was mainly perceived by women aged ≥ 60 years and who had people at home with high-risk medical conditions or aged ≥ 65 years. It was also observed that the higher the perceived susceptibility and severity of COVID-19, the higher the fear level. Moreover, the fear level was higher among women whose perception was that people were insensitive to the pandemic and ignoring its risks.

### 4.2. Lifetime and risk perception

The findings of this study revealed some characteristics related to the women’s age. Younger women (18–29 years) exhibited a high odds of perceiving themselves as highly susceptible to COVID-19 infection (Table 3); women aged 30–59 showed a higher perceived impact on daily activities in case of infection than those aged 18–29 years (OR = 1.34); and the perceived severity of COVID-19 (referring to the threat to life posed by COVID-19) was higher among women aged 60 years and above (Table 5). An attempt to explain these results stems from the social roles and the particular conditions related to the moment of life. The three results are associated with age groups, which, in turn, correspond to experiential factors within sociocultural references.

In our study, younger women who perceived themselves as at high risk of being infected may have maintained paid work activities outside the home. Besides, given the high family demand (Power, 2020) due to circulation restrictions (especially for family members with comorbidities and the elderly), they probably had to meet their needs, taking responsibility for essential activities outside the home, such as going to the market, bank, and pharmacy, or accompany other family members to medical services. Thus, there may be a correspondence between maintaining circulation outside the home in public spaces and perceiving that this increased circulation also leads to greater exposure to the virus.

Women aged 30–59 years also perceived a high impact on daily activities if infected with COVID-19. It can be attributed to its triple burden of responsibilities: participation in the productive and reproductive economy and in the emotional care of the family (Chung, 2020; Chung & van der Lippe, 2020; Power, 2020). In addition to their professional responsibilities, women usually exercise a prominent position in the family sphere, especially in the domestic routine and emotional care of family members. Thus, it is plausible to assume that married women with children and professional careers can foster a feeling that whether their health was affected, they would not be able to maintain all of their activities effectively. This overload of domestic work ‒ historically done by female hands (Moreira da Silva, 2019) and which increased considerably during the pandemic due to the whole family being at home (Hipp & Bünning, 2021; ILO, 2020; Power, 2020; Reichelt, Makovi, & Sargsyan, 2021) ‒ may be amongst the factors associated with the particular way in which women are experiencing this unique moment in different societies, including the Brazilian one.

About older women, the high perceived severity of COVID-19 (referring to the threat to life posed by COVID-19) among those aged 60 and above is an understandable feeling in a group characterized as the most susceptible to develop severe illness from COVID-19. However, the media strategy used to publicize the severity of COVID-19 based on individual characteristics of greater biological vulnerability (such as age, chronic illness, and pregnancy), in order to sensitize these individuals to the need for self-care, has the side effect of stigmatizing these groups and producing feelings of fragility (Matta et al., 2020). The feeling of vulnerability is possibly amplified due to the Brazilian government’s absence of a planned and coordinated response that evokes confidence in the public institutions. The political ideology based on the negationism adopted by the federal government concerning the pandemic, added to the daily numbers of deaths and collapse of the health system, may have contributed to the high level of fear, high-risk perception, and feeling of insecurity in the population (Ribeiro, Lima, & Waldman, 2020; The Lancet, 2020). Research by Dryhurst et al. (2020) revealed that higher confidence levels in the government are associated with low-risk perception.

### 4.3. Gender and socio-epidemiological inequalities

The aspects of the pandemic’s impact on the female gender with peculiarities in certain groups need to be understood based on the socio-epidemiological inequalities that characterize the country and the socio-epidemiological characteristics of the lifetime related to gender social roles.

In 2020, a woman was murdered in Brazil every two hours, with 38.9% of femicides occurring at home by intimate partners (IPEA, 2020). In the pandemic, from March to April 2020, homicides of women increased by 22.2% compared to the same period in 2019 (CNN, 2020).

The life expectancy of Brazilian women is approximately 80 years, with women over 25 years of age studying on average only 9.6 years (IBGE 2019). This low educational attainment is associated with other indicators, such as the average age at birth of the first child and marriage. In addition, the highest marriage rates occur among younger women, with 27 years being the average age at first marriage. In 2016, 35.6% of the female population married before the age of 18 in Brazil (IBGE, 2020).

Regarding the age pattern of fertility, Brazil has a rejuvenated fertility pattern similar to the Latin American pattern, characterized by the highest fertility at young ages. However, the country has a significant difference in the average number of children per woman when considering education, economic situation, and the country’s region. This average is lower among those with higher education, better financial conditions, and residents in the south and southeast regions than among those with lower education, worse financial conditions, and residents in the northern region. Nevertheless, the country still has only 10% of women without children (mainly among those with higher education), which is relatively lower than in European countries where this percentage varies from 20 to 25% (UNFPA, 2018).

In Brazil, children’s presence at home is directly related to women’s lower labor market insertion. The national study ‘Gender Statistics’ carried out by the Brazilian Institute of Geography and Statistics revealed that the presence of children up to three years of age at home is an important feature in determining women’s occupation in the labor market. In 2019, the occupation proportion was 54.6% among women who had children of this age against 67.2% among those who did not (IBGE, 2020). In addition, the greater involvement in unpaid work contributes to explain the lower participation of women in the labor market. In 2019, women also dedicated nearly twice as much time per week as men to caring for people and doing household chores (21.4 h against 11.0 h) (IBGE, 2020).

Although Brazilian women generally study for more years than men, more than half aged ≥ 15 years are part of the country’s workforce, and almost half of the households are financially maintained by women; they receive just over three quarters (77, 7%) of men’s wages (IBGE, 2020).

Since the beginning of the pandemic, Brazilian women were impacted by schools and daycare centers’ closure. In the absence of coping measures, with emergency public policies in the education area, social or labor policies aimed at maintaining female jobs and social assistance to women who lost their jobs, the rate of adverse effects concerning sex and gender was disproportionate (ILO, 2020; Reichelt et al., 2021). Since then, many women have precariously exercised their work activities and assumed a triple workload, taking care of their children’s education (United Nations, 2020) and domestic chores (like cleaning the house and preparing the family’s food), along with paid work activities (formal or informal) (Chung & van der Lippe, 2020; Hipp & Bünning, 2021).

### 4.4. The gender of the care economy and risk perception

As previously noted, the perceived susceptibility to COVID-19 infection was higher among women who had children under the age of ten and those who had people at home with high-risk medical conditions for severe illness from COVID-19. These characteristics are related to the care and responsibility for the loved one’s life, a typical role of the female gender.

Lagarde (2005) pointed out that a gender condition for women is the requirement to donate and dedicate life to others (as a mother or wife). Therefore, the burden of household chores and the care of family members automatically falls on women. In this space, the gender stereotype is realized by attributing completeness and female fulfillment in care and motherhood (Giordani, Piccoli, Bezerra, & Almeida, 2018). In the pandemic context, it is then up to the woman to worry about whether her family members will not be contaminated and take care of them if they become infected. Thus, it can contribute to higher levels of fear and a higher perception of risk since concerns are focused on individual and family well-being.

Clearly, women express a higher perception of the vulnerability of life in the face of the threat of the virus, which manifests itself in its central role and activities to meet the physical and emotional demands and the family’s well-being. Some researchers call this the economics of care: an emotional work that aims to ensure family members’ well-being and represents a mental burden of concern invisible by unequal relations between genders (Chung, 2020; Power, 2020).

Especially in the reproductive period, women tend to experience great vulnerability, which can be associated with the environment and social relationships. For example, research carried out in Ethiopia, India, and Vietnam revealed that mothers of small children who experience stressful family-related life events, such as illness or death within the household and financial uncertainty, are more likely to experience future severe mental distress episodes (Gausman & Langer, 2020). Complementing this argument to understand women’s situation in the pandemic, Hamadani et al. (2020) identified that the stay-at-home orders (lockdown) worsened women’s health who lived in rural Bangladesh. It also exposed them to increased domestic violence, moderate and severe food insecurity, and financial instability, resulting in increased depression and anxiety symptoms.

Studies have shown how the emotional experience of the pandemic can have different nuances for men and women. For example, studies using the FCV-19S have shown higher levels of fear among women than among men. In a study carried out in Cuba by Broche-Pérez, Fernández-Fleites, Jiménez-Puig, Fernández-Castillo, and Rodríguez-Martin (2020), gender was significantly associated with fear of COVID-19 and psychological vulnerability during the pandemic. In such a study, being female was a predictor of medium and high levels of fear of COVID-19. Compared to the low level of fear, women relative to men showed an increase of 213% and 245%, respectively, in the odds of reporting medium and high fear levels. In our study, the overall mean score of fear of Brazilian women (20.4, SD = 5.2) was slightly higher than that of the general Brazilian population (19.8, SD = 5.3) (Giordani, Zanoni da Silva, Muhl, & Giolo, 2020) and lower than that reported for the Cuban female population (21.9, SD = 6.9) (Broche-Pérez et al., 2020). Research by Dryhurst et al. (2020) also found an association between female gender and perception of high susceptibility to infection.

Recognizing that ‘gender’ contains sociocultural and psychological attributes allows us to understand how each woman synthesizes and realizes in her own lived experience the gender configurations of her society, which, in turn, generate specific ways of coping and resource mobilization. The findings of our study demonstrated how women’s life circumstances constitute a particular condition in the world. This was essential to analyze risk as a perception of negative results and fear as an emotion linked to risk experience.

### 4.5. Study limitations

Finally, this study has some limitations. First, it is a cross-sectional study based on a non-probabilistic sampling; thus, it is difficult to establish causality due to the study design. Second, the results are restricted to the population strata covered by the sample: women with better economic conditions and higher education, which do not represent Brazilian women’s average profile. Concerning these two limitations, it should be noted that the need to access the internet and electronic devices (cell phone, notebook, and tablet) to answer the survey might have hindered the participation of other sociodemographic profiles in the current study. Third, mistakes in the answers can result from how the data were collected: through electronic self-completion and without the presence of an interviewer to clarify doubts. Fourth, the effects of volunteering recruitment include a sample of participants motivated by interest and sensitivity to the topic, causing participation bias.

It is recommended that future studies investigate mixed samples to deepen the understanding of the differences between men and women and also investigate male samples that elucidate the specifics of the pandemic risk experience for men. Longitudinal studies of the FCV-19S would also provide important information about how the fear of COVID-19 is changing with changes in infection and death rates.

## 5. Conclusion

The pandemic’s social and economic consequences, combined with gender inequalities that already exist in Brazil, will probably amplify the damage of the effects of the crisis on women’s lives. Thus, investigating how women experience the COVID-19 pandemic in Brazil is a way of getting to know the particularities of gender configurations in the country.

Our study identified specific characteristics such as age group and living conditions (especially those related to family and domestic life) associated with higher levels of fear, greater perceived susceptibility to infection, and greater perceived severity of COVID-19. The United Nations (United Nations, 2020) estimates that the pandemic and its consequences should disproportionately affect the most vulnerable groups, including women. Moreover, the potentially adverse effects on health, well-being, and women’s productive lives may even last longer than men. Therefore, these inequalities must be the object of critical analysis to support public policies based on evidence capable of mitigating the pandemic’s consequences and also preventing and overcoming inequalities between women and men.

## Data Availability

This article includes the data set collected in the study and the respective codebook. They can be uploaded from the Figshare repository doi:10.6084/m9.figshare.13542989.
